# Dose-dependent impact of oxytetracycline on the veal calf microbiome and resistome

**DOI:** 10.1186/s12864-018-5419-x

**Published:** 2019-01-19

**Authors:** Bart J. F. Keijser, Valeria Agamennone, Tim J. van den Broek, Martien Caspers, Adri van de Braak, Richard Bomers, Mieke Havekes, Eric Schoen, Martin van Baak, Daniël Mioch, Lonneke Bomers, Roy C. Montijn

**Affiliations:** 1 Research Group Microbiology and Systems Biology, TNO, Utrechtseweg 48, 3704 HE Zeist, The Netherlands; 2 DUCARES B.V, Reactorweg 47-A, 3542 AD Utrecht, The Netherlands; 3 Dierenartsenpraktijk Vaassen, Laan van Fasna 18, 8171 KH Vaassen, The Netherlands; 4 Department of Risk Analysis of Products in Development, TNO, Utrechtseweg 48, 3704 HE Zeist, The Netherlands; 5 SKV, Nevelgaarde 20d, 3436 ZZ Nieuwegein, The Netherlands

**Keywords:** Antibiotic, Antibiotic resistance, Veal calves, Gut microbiome, Resistome, Oxytetracycline, Metagenome, Minimum selective concentration, Sub-therapeutic concentration

## Abstract

**Background:**

Antibiotic therapy is commonly used in animal agriculture. Antibiotics excreted by the animals can contaminate farming environments, resulting in long term exposure of animals to sub-inhibitory levels of antibiotics. Little is known on the effect of this exposure on antibiotic resistance. In this study, we aimed to investigate the long term effects of sub-inhibitory levels of antibiotics on the gut microbiota composition and resistome of veal calves in vivo.

Forty-two veal calves were randomly assigned to three groups. The first group (OTC-high) received therapeutic oral dosages of 1 g oxytetracycline (OTC), twice per day, during 5 days. The second group (OTC-low) received an oral dose of OTC of 100–200 μg per day during 7 weeks, mimicking animal exposure to environmental contamination. The third group (CTR) did not receive OTC, serving as unexposed control. Antibiotic residue levels were determined over time. The temporal effects on the gut microbiota and antibiotic resistance gene abundance was analysed by metagenomic sequencing.

**Results:**

In the therapeutic group, OTC levels exceeded MIC values. The low group remained at sub-inhibitory levels. The control group did not reach any significant OTC levels. 16S rRNA gene-based analysis revealed significant changes in the calf gut microbiota. Time-related changes accounted for most of the variation in the sequence data. Therapeutic application of OTC had transient effect, significantly impacting gut microbiota composition between day 0 and day 2. By metagenomic sequence analysis we identified six antibiotic resistance genes representing three gene classes (tetM, floR and mel) that differed in relative abundance between any of the intervention groups and the control. qPCR was used to validate observations made by metagenomic sequencing, revealing a peak of tetM abundance at day 28–35 in the OTC-high group. No increase in resistance genes abundance was seen in the OTC-low group.

**Conclusions:**

Under the conditions tested, sub-therapeutic administration of OTC did not result in increased tetM resistance levels as observed in the therapeutic group.

**Electronic supplementary material:**

The online version of this article (10.1186/s12864-018-5419-x) contains supplementary material, which is available to authorized users.

## Background

Antibiotic therapy is commonly used in animal agriculture both to treat ill animals and as a form of metaphylaxis, to prevent the development and spreading of infections in high-risk conditions. Low doses of antibiotics can also be added to animal feed to promote growth, although this practice has been banned in the European Union since 2006 [1]. The use of antibiotics can lead to the selection of resistant strains in livestock, shedding in the food chain and posing a risk for food safety and human health [2]. Furthermore, resistant strains, along with residual antibiotics, antibiotic metabolites and antibiotic resistance genes, eventually can reach waters and soils [3]. As a result, bacteria in these environments are frequently exposed to low concentrations of antibiotics, which can impact microbial ecosystems through community shifts and alterations of the resistome [4, 5].

Under laboratory conditions, it has been shown that selection for antibiotic resistance can occur even at very low antibiotic concentrations [6]. Selection mechanisms for resistance at sub-inhibitory concentrations are different from those active in the presence of lethal antibiotic concentrations [7]. Exposure to very low concentrations of antibiotics – even below the minimum inhibitory concentration (MIC) – elicits an adaptive resistance response [8], and can result in the presence of a wider range of resistant mutants, increased genotypic [9] and phenotypic [8] diversity, and higher rates of mechanisms that allow spreading of resistance, such as horizontal gene transfer (HGT) [10]. It has also been shown that resistance mutations in bacteria exposed to sub-MIC antibiotic concentrations are different from those of bacteria exposed to high concentrations [11], supporting the presence of alternative resistance mechanisms.

Many aspects of the development of antibiotic resistance in complex microbial ecosystems encountered in vivo in the presence of low concentrations of antibiotics still need to be elucidated. While pure culture laboratory studies allow detailed analysis of the impact of antibiotics under well controlled experimental conditions, such studies lack the complex ecological interactions encountered in vivo. Competition between microorganisms, host-microbe interactions and the impact of environmental conditions (including food/feed intake) exert ecological pressure, and may influence the net effect of antibiotics, especially at low concentrations.

The main objective of this study was to assess the effects of a long term exposure to a low dose of antibiotics on the veal calf gut microbiome in vivo. We performed an intervention study where we administered oxytetracycline to calves, either at a high therapeutic dose (oral therapy for 5 days), or at a low dose, to mimic exposure to environmental contamination. We then used a combination of methods (antibiotic analysis by the UPLC-MS/MS, 16S ribosomal sequencing, shotgun sequencing, quantitative PCR) to determine the effects of the interventions on the composition of the fecal microbiota of the calf and on the presence of antibiotic resistance genes therein.

## Methods

### Experimental design: Animals, treatment and sample collection

Farm-level studies were performed between 9 − 6-2015 and 28-7-2015 accordance with the Dutch Law on Animal Health and Welfare. Sixty healthy male Friesian Holstein calves (between 2 and 4 weeks of age), bodyweight approx. 50 kg, were collected from different dairy farms in in the regions Nordrhein-Westfalen, Rheinland-Pfalz, Saarland, Thüringen, Sachsen and Hessen (Germany) and transported via a calf-collecting-center in Nordrhein-Westfalen (Germany) to a veal farm where they were housed individually in indoor pens in a clean, newly built barn. Animals were placed under regular agricultural care according to the regulations of the Integrated Chain Management System (IKB) and regular clinical veterinary practices. Following a 1 week period during which the calves adapted to the new environment and were fed according to routine, the intervention was initiated. Of the 60 calves, 42 calves that were shown to have fecal antibiotic levels upon arrival below 100 μg/kg feces were selected and assigned to three intervention groups. Each intervention group consisted of 14 animals, housed in such a way as to separate the control and low dose group from the therapeutic level intervention group. In addition, contact between calves was only possible with neighboring animals of the same experimental group (Additional file 1: Figure S2). Logistics in the barn were organized in such a manner that it would minimize carriage of antibiotic residues within the barn. In addition all materials required for animal treatment were antibiotic free and were available in separate color coded sets for each of the three groups. The first group (OTC-high) received an oral dose of 1 g OTC in two liters Calf Milk Replacer (CMR), administered twice a day for 5 days. The second group (OTC-low) received an oral dose of OTC of 25 μg/L CMR daily during 7 weeks (increasing from 100 μg/day at the start of the intervention until 200 μg/day at the end), mimicking animal exposure to environmental contamination [12, 13]The third group (control) did not receive OTC, serving as a unexposed control (Additional file 2: Figure S1). The three groups are referred to in the paper as OTC-high, OTC-low and CTR respectively. Calves were fed antibiotic-free CMR twice daily throughout the trial. The amount of CMR increased from four liters per day at day 0 to eight liters per day at day 42. The CMR was supplemented with etheric oil solution (Bronch-Arom F) and linseed oil. The calves also received roughage (starting with 50 g/d, rising to 450 g/d) and a homeopathic preparation throughout the trial, and they were on individual basis supplemented with Globatan, vitamin E and Vitamix.

On three occasions, individual treatment using non-antibiotic supplements did not sufficiently relieve respiratory symptoms. An antibiotic treatment was therefore required upon indication by the responsible veterinarian. In accordance with the study protocol, non-tetracycline antibiotics were given to all animals in order to avoid any bias between the study groups. Three days prior to the start of the study, all calves were given 600 mg tilmicosin (Milbosin, Dechra). At day 5, 1500 mg Florfenicol (NuFloR, MSD) was administered. At day 21, tilmicosin (Milbosin) was again given to all animals at a dose of 900 mg. Milbosin was given by subcutaneous injection. NuFlor treatment was done by intramuscular injection. Treatment was successful and relieved animals from respiratory symptoms and prevented further infection among the flock.

Fecal samples were collected within 24 h after arrival (day − 6), at the start of the intervention (day 0), and at days 2, 6, 14, 21, 28, 35 and 42. The fecal samples were cooled upon collection, and shipped to the laboratory where they were aliquoted and snap frozen in liquid nitrogen and stored at − 80 °C. The time between sampling and freezing was at most 4 h.

### Antibiotic residue analysis

After thawing, homogenized feces (1 ± 0.05 g) were weighed in a 50 mL polypropylene test tube. Samples were spiked with an internal standard of antibiotic isotope variants as appropriate and mixed. The antibiotics were extracted from the feces using 10 mL methanol:acetronitril:0.1 M EDTA-McIlvainbuffer (pH 4.0), (30:20:50 ^*v*^/_v_ %) and 15 min shaking. After extraction the samples were centrifugated at 3800 x *g* for 15 min. The organic phase of the supernatant was evaporated under a stream of nitrogen at a temperature not exceeding 45 °C. After this the extraction was repeated, and the two supernatants were combined, 27 mL 0.2 M Phosphate buffer (pH 6.5) was added. Further clean-up was performed on Oasis HLB cartridges (200 mg, 6 cc, WATERS). 5 mL of the supernatant was passed through the columns. After elution the extracts were evaporated to dryness under a stream of nitrogen at a temperature not exceeding 45 °C. The residue obtained was dissolved in 500 μL 0.1% HCOOH in H_2_O/MeOH (1:3 ^v^/_v_ %) for injection into the UPLC-MS/MS system. Analysis was performed on a Sciex 6500 QTRAP mass spectrometer (Sciex, Massachusetts, USA) connected to an Agilent 1290 Infinity (Agilent, California, USA) LC system. The mass spectrometer was operated in a positive and negative electrospray ionisation (ESI^+^ and ESI^−^) mode. The desolvation temperature was 400 °C and the cone temperature was 120 °C. Over 100 analytes were analysed in scheduled multiple reaction monitoring (SMRM) mode. Two transitions per analyte were created. The chromatography was performed on an Acquity UPLC BEH C18, 2.1x100mm, 1.7 μm column (WATERS, Massachusetts, USA). A gradient containing 0.1% formic acid (A) and 0.1% formic acid (HCOOH) in MeOH (B) was applied. The gradient went from 95% A stepwise to 100% B. The runtime for each injection was 12.5 min, the flow 0.4 mL min^− 1^ and the injection volume was 5 μL. Between runs, calibration samples were run composed of a mixture of 46 antibiotics.

### DNA extraction and qPCR

For DNA isolation, fecal samples were thawed on ice and lysed by bead beating (Mini-BeadBeater-24, Biospec Products Bartlesville, USA) for 2 min at 2800 oscillations minute^− 1^ in the presence of 300 μl of lysis buffer (Mag Mini DNA Isolation Kit, LGC ltd, UK), 500 μL zirconium beads (0.1 mm; BioSpec products, Bartlesville, OK, USA) and 500 μL phenol saturated with 10 mM Tris-HCl and 1 mM EDTA pH 8.0 (Sigma). After centrifugation DNA was extracted using the Mag Mini DNA Isolation Kit (LGC ltd, UK) in accordance to the manufacturers recommendations. DNA quality was assessed by routine gel electrophoresis as well as by capillary electrophoresis on the Fragment Analyzer (Advanced Analytical, Heidelberg, Germany). Quantitative PCR (qPCR) primers and probes are listed in Additional file 3: Table S1. qPCR was performed using RT PCR master mix (Diagenode, Seraing, B) on an Applied Biosystems 7500 RT PCR system.

### High-throughput sequencing

For 16S rDNA amplicon sequencing of the V4 hypervariable region, 1 ng of DNA was amplified as described by Kozich et al. [14] with the exception that 33 cycles were used instead of 35, using F515/R806 primers [15]. As control, mock samples comprising a mixture of 24 pure culture isolates, blanco extraction controls and two pooled fecal samples were included in each batch. Primers included the Illumina adapters and a unique 8-nt sample index sequence key [15]. The amount of DNA per sample was quantified using the Quant-iT™ PicoGreen® dsDNA Assay Kit (Thermo Fisher Scientific). The amplicon libraries were pooled in equimolar amounts and purified using the IllustraTM GFXTM PCR DNA and Gel Band Purification Kit (GE Healthcare, Eindhoven, The Netherlands). Amplicon quality and size was analysed on the Fragment Analyzer (Advanced Analytical). Paired-end sequencing of amplicons was conducted in five separate runs on the Illumina MiSeq platform (Illumina, Eindhoven, The Netherlands). The sequence data was processed with Mothur v.1.31.2 [16], in line with the mothur MiSeq SOP [14]. Sequences were grouped in operational taxonomic units by Minimal Entropy Decomposition (MED) using a minimum substantive abundance value (−M) of 500 [17]. Taxonomy was then assigned by querying the representative sequence of each oligotype against the SILVA database (release 132) [18].

Metagenomic shotgun sequencing (125 nt paired end sequencing) was performed at Baseclear (Leiden, The Netherlands) on the Hiseq2500. After quality filtering and removal of host genes, metagenomic sequence reads were mapped against the antibiotic resistance gene database CARD [19] (libraries AR, AT and ABS) using mapping software bowtie2 [20](applying method = −-very-fast-local). This allowed enumeration of reads/resistome element in each sample. Reads were allowed to map on multiple resistome genes (multiple mapping) in order to obtain maximal sensitivity to detect resistome elements. Mapping frequencies were normalized by using the total number of reads per fecal sample. An abundance filter was applied in which each resistance gene was required to have at least 3 sequence reads in at least four animals in at least one group at one timepoint. Metagenomic shotgun sequences were mapped against the non-redundant protein database through BLASTX using DIAMOND (V.0.9.9) [21]. Mapping results were parsed in MEGAN (v. 6) [22] and linked to KEGG functional modules (KEGG reference library december2017) [23].

### Statistical analysis of 16S rRNA amplicon and metagenomic sequencing data

All statistical analyses on the 16S sequencing data were performed using R version 3.3.2 [24]. The overview of all sample groups in Fig. 2 was creating using classical multidimensional scaling as implemented in R ‘base’. A multidimensional scaling procedure was applied to a Bray-Curtis dissimilarity matrix created using the ‘dist’ function of the ‘proxy’ package [25]. All distance displayed in the boxplots of Fig. 3 were extracted from the same distance matrix as the one used for the multidimensional scaling procedure. Statistics on the distances shown in Fig. 2 were performed using linear mixed models with Tukey HSD post-hoc testing (no *P*-value correction), as implemented in the packages ‘lme4’ and ‘multcomp’ respectively [26, 27]. For the linear mixed model, the *subject* was used a random factor. Multivariate statistics on the 16S sequencing data was performed using PERMANOVA and constrained analysis of principal coordinates as implemented in the ‘vegan’ package [28]. Shannon diversity was calculated using the ‘vegan’ package. The diversity measures were transformed to deal with non-normality using Box-Cox transformation (as implemented in the ‘caret’ package [29]) and subsequently analyzed for statistical differences using linear mixed models with the subject as a random effect.

The KEGG module count data derived from the metagenomic shotgun data were analysed using DESeq2 [30], comparing differences in relative abundance of KEGG modules between groups at day 6 and day 42.

### Statistical analysis of resistome

For each timepoint, the abundance of each gene as a function of the experimental group was modeled using a Poisson regression model with overdispersion [31]. The abundance was normalized for the total number of reads in each sample. The model specification is as follows:

$$ {Y}_{ij}\sim P\left({\mu}_{ij},\varPhi \right), $$

where Y denotes the abundance, i denotes the experimental group (1 = control, 2 = high, 3 = low), j denotes the animal in a group (j = 1…9), is the true value of the abundance, P denotes a Poisson distribution with overdispersion, and Φ denotes the dispersion parameter.

The true value of the abundance is related to the experimental conditions by

$$ \log {\mu}_{ij}=C+{\varDelta}_i+\log \raisebox{1ex}{${h}_{ij}$}\!\left/ \!\raisebox{-1ex}{$H$}\right. $$

where C is the level of control group, Δ_i_ is the increase of group i compared to the control, h_ij_ denotes the total number of reads of animal j in group i, and H denotes the largest number of reads in all 81 samples.

For each time point and gene, the Poisson regression model results in a *P* value that assesses whether the experimental groups differ in abundance. We applied a multiple test correction on all the *P* values [32]. The genes whose FDR-corrected P values for the test on group differences were smaller than 10% for at least one time point were studied further with t tests comparing the respective intervention groups with the control group.

### Data deposition

The datasets supporting the conclusions of this article are available in the Sequence Read Archive (SRA) repository, project ID: PRJNA511412; https://www.ncbi.nlm.nih.gov/bioproject/PRJNA511412.

## Results

### Intervention

To investigate the effects of therapeutic and environmental oxytetracycline exposure on the calf fecal microbiome and resistome, an intervention study was performed. After a run-in period of 7 days, 42 calves of 2–4 weeks of age were randomly assigned to three groups. The first group (OTC-high) received therapeutic oral dosages OTC, of two grams daily during 5 days. The second group (OTC-low) received a daily oral dose of 100 μg/day and increasing to 200 μg/day during 7 weeks, mimicking animal exposure levels to environmental contamination, as observed in previous studies [5, 12, 13, 33–36]. The third group (CTR) did not receive OTC, serving as a unexposed control. Animals were housed in individual pens, isolating the control and low dose group from the therapeutic level intervention group.

### OTC levels discriminate between the intervention and control groups

**Fig. 1:**
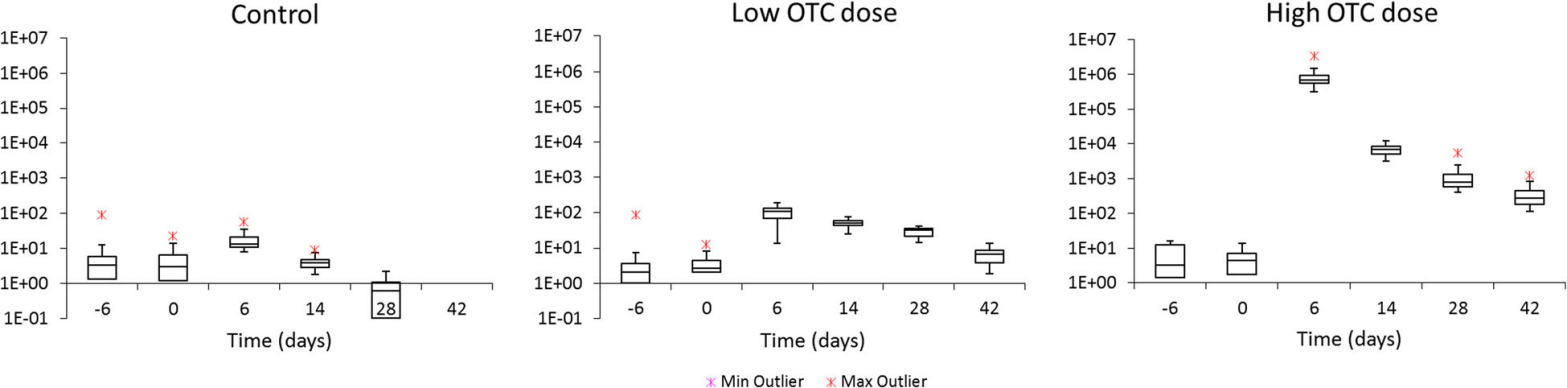
OTC levels (μg/kg) measured at different time points in fecal samples from calves from the control, the low-dose, and the high-dose groups

Fecal antibiotic residue levels were determined by UPLC-MS/MS techniques. OTC, tetracycline, tilmicosin, tylosin and florfenicol were the only antibiotics detected to levels greater than 5 μg/kg in the fecal samples taken during the intervention period. The low levels and time of occurrence for tetracycline and tylosin were in correspondence with their likely origin as contaminants in the veterinary medical products used of OTC and tilmicosin administration. The levels of fecal OTC clearly discriminated the three groups (Fig. 1). The therapeutic intervention group (OTC-high) showed a marked increase in fecal OTC levels immediately upon initiation of the treatment which declined to lower levels during the intervention. The median fecal OTC levels in the OTC-high group increased at day 2 to 335 mg/kg, and 682 mg/kg at day 6. At subsequent timepoints, the fecal OTC levels declined by approximately 90% each week. For the group receiving a continuous oral dose of OTC of 25 μg/L calf milk replacer (CMR), the median fecal levels of OTC were highest at day 6 at 111 μg/kg, declining to 8 μg/kg at day 42. Measurable fecal levels of OTC were detected in the control group, with highest median levels of 13 μg/kg at day 6. OTC fecal levels declined to 3 μg/kg at day 14 and < 2 μg/kg at day 28. In following time points no detectable levels of OTC was measured in feces. A marked consistency was found in the fecal OTC levels between calves within each group. In those samples in which higher levels of OTC was detected, we also found low levels of tetracycline. Tilmicosin was found in all three study groups, showing highest levels at day 0 and day 28, in line with application at day − 3 and day 21. Low levels of tylosin were detected in those samples in which highest levels of tilmicosin was found. Florfenicol was found in low but detectable levels (< 5 μg/kg) at day 14, probably as a residual trace of Florfenicol application at day 5.

### Time and not antibiotic levels related most of the observed variation in microbiota composition

**Fig. 2:**
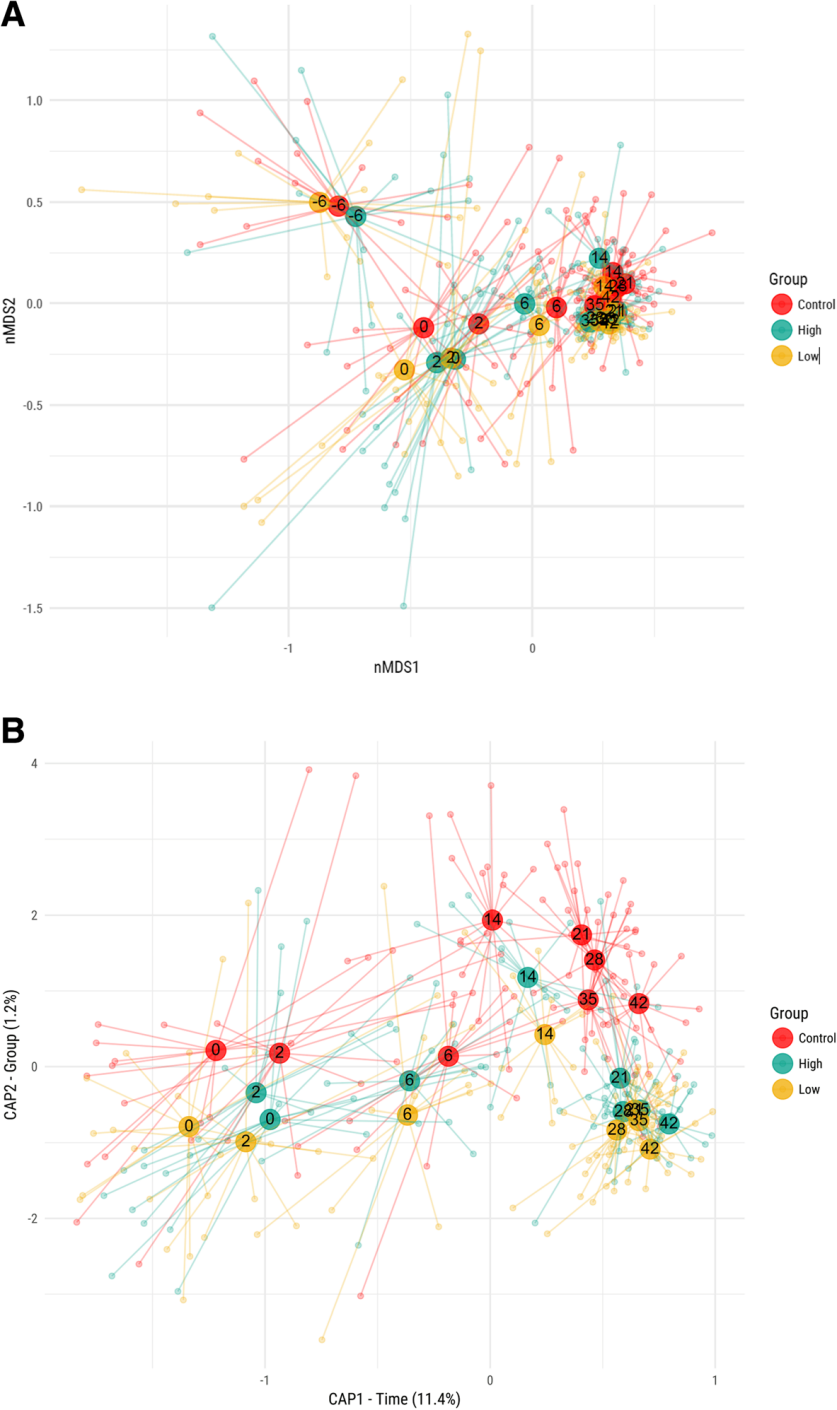
**a** Multidimensional scaling (MDS) plot of microbial community dissimilarities based on 16S rRNA gene sequencing data. The plot shows the relationships between the intervention and control groups. Samples are shown as translucent dots; the annotated opaque circles represent the centroids of each of the sample groups at the respective time points. **b** Canonical Analysis of Principal coordinates (CAP) of microbiota composition data. Analysis was constraint for time (x-axis) and group (y-axis)

The fecal microbiota composition was analyzed for all calves at all time points by 16S ribosomal amplicon sequencing. In total 23,8 million raw sequence reads were generated, averaging 43.017 (± 19.742) sequences per sample. After subsampling at 8.734 sequences, 606 operational taxonomic units were generated by minimum entropy decomposition, retaining a total of 3.4 million processed sequences (Additional file 4: Figure S3). To visualize the microbiota community development for the different groups over time we used multidimensional scaling (MDS) (Fig. 2A). The non-metric MDS plot showed that the largest variation (nMDS-1) in the microbiota composition was related to a time-dependent development. After day 14, the fecal microbiota community structure appeared to have stabilized, resulting in overlapping centroids of samples taken between day 14 and day 42. The second largest variation (nMDS-2), appeared to relate to shifts in microbiota community structure during the run-in period between day − 6 and day 0, linked to lowered *Bacteroides* levels and increased levels of *Lactobacillus*, *Bifidobacterium* and *Blautia* species. From the MDS plot, we did not see a clear separation between the control and the antibiotic intervention groups. To visualize possible differences related to the intervention groups, separated from temporal changes, we used Canonical Analysis of Principal coordinates (CAP), constraining for ‘time’ (x-axis) and ‘group’ (y-axis) (Fig. 2B). In the analysis we excluded samples from day − 6 to avoid a bias related to changes during the run-in period. This analysis showed that 11.4% of the variance could be explained by temporal changes between baseline (day 0) and day 42, revealing a similar temporal pattern for all groups. The variance linked to ‘group’ differences was 1.2%, and was mainly related to a separation of the control group and the two intervention groups (high and low dose) at later time points. Microbiota changes related to time, as identified by examining the coefficients of the CAP model defining the time axis suggested a decrease in *Alloprevotella, Bifidobacterium, Faecalibacterium,* and *Streptococcus* species and increasing levels in *Peptococcus, Prevotella* and *Bacteroidales* species with increasing age (Additional file 5: Figure S4A). Microbiota changes related to groups related to higher le,vels of *Ruminococcus, Coprobacillus and Lachnospiraceae species* and lower levels of *Prevotella*, *Faecalibacterium* and *Blautia* species in the control group compared to the low and high dose intervention groups (Additional file 5: Figure S4B). Bacterial diversity (Shannon diversity) showed an increase over time, but showed no apparent difference between groups upon completion of antibiotic administration in the high group (day 6), nor upon completion of the study (day 42) (Additional file 6: Figure S5).

We used permutational multivariate analysis of variance (PERMANOVA) to examine the significance of differences in microbiota community composition between intervention groups at each time point (Table 1). With the exception of day 0 and day 14, the control and the OTC-high group were significantly different at each time point (*p* < 0.05 at days 2 and 6; *p* < 0.005 at days 21, 28, 35 and 42). The control and the low group were significantly different at day 21 through 42 (p < 0.05). The low and the high groups were significantly different at day 2 and 6 (p < 0.05) and at day 42 (p < 0.005).

**Table 1:** *P*-values derived from PERMANOVA pairwise statistical comparisons of microbial community composition between groups at each time point. Grey boxes indicate *p* < 0.05; grey boxes with bold text indicate *p* < 0.005

	Day 0	Day 2	Day 6	Day 14	Day 21	Day 28	Day 35	Day 42
control - low	0.2699	0.8024	0.8024	0.2587	**0.0013**	**0.0036**	0.0146	**0.0013**
control - high	0.7038	0.0288	0.0288	0.6259	**0.0013**	**0.0036**	**0.0047**	**0.0013**
low - high	0.6847	0.0428	0.0428	0.0684	0.2023	0.0675	0.3031	**0.0013**

**Fig. 3:**
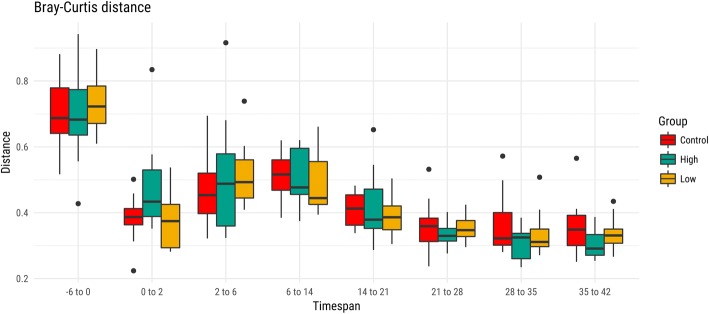
Boxplots showing the Bray-Curtis dissimilarities between individual calves within each group, for pairs of subsequent time points. The boxes in the plot represent the interquartile ranges, the horizontal lines give the position of the medians, the vertical bars indicate the range. The dots indicate outliers

We also applied PERMANOVA to assess the significance of differences in microbiota community composition within groups between subsequent timepoints (Table 2). The analysis showed significant changes within the control group during the first 2 weeks (*P* < 0.05) but not in the succeeding time points. For the OTC-high as well as the OTC-low dose group, significant changes were detected at each subsequent time interval throughout the study (P < 0.05). The microbiota differences supporting the PERMANOVA models were in full agreement with the coefficients defined in the Canonical Analysis of Principal coordinates (CAP) analysis related to temporal changes. We quantified the change in fecal microbiome composition for the individual calves at sequential time points by calculating the Bray-Curtis distances (Fig. 3). This confirmed the relative large community shifts between day − 6 and day 0 and the stabilization of the microbiota composition with increasing age, as noted by the smaller distances between subsequent time points and smaller variation between calves. We found a significantly (P < 0.05) larger Bray Curtis distance between day 0 and day 2 for calves in the OTC-high group compared to the other groups, indicating that within this timeframe a significantly larger change in the microbiome occurred in the OTC-high group compared to other groups. Between day 2 and day 6, the Bray Curtis dissimilarities of the OTC-high group appeared to be larger than in the other groups, but this was not statistically significant. No significant differences were detected in Bray Curtis distances between groups at subsequent time points.

**Table 2:** P-values derived from PERMANOVA pairwise statistical comparisons of microbial community composition within groups between subsequent time point. Grey boxes indicate p < 0.05; grey boxes with bold text indicate p < 0.005

	-6-- > 0	0-- > 2	2-- > 6	6-- > 14	14-- > 21	21-- > 28	28-- > 35	35-- > 42
control	**0.0013**	0.3584	0.0103	**0.0069**	0.1938	0.1335	0.2699	0.0428
high	**0.0013**	0.1066	**0.0091**	**0.0013**	0.0024	0.0113	0.0266	0.0013
low	**0.0013**	0.627	0.0769	**0.0013**	0.0024	0.0069	0.0534	0.0047

### Functional differences discriminate both intervention groups from the control group

We performed metagenomic shotgun sequencing of feces samples taken at baseline (day 0), day 6 and day 42, to explore potential functional differences in the fecal microbiota between the intervention groups. To facilitate the comparison, we aggregated the shotgun reads to the level of functional gene orthologous, using the KEGG Orthology, and we used DESeq2 to reveal significant differences between intervention groups. Table S2 lists the orthologous groups with significantly different abundances between groups (*p* < 0.01). Most functional differences were detected between groups at day 42. At day 6, one gene orthologue (K19172) was found to be enriched in the high group compared to the control and low group. This orthologue group was found to be depleted in the high group compared to the control and low groups at day 42. K19172 consists of DndE, DNA sulfur modification proteins [37]. No other functional differences were detected between groups at day 6. The most profound difference detected at day 42 was the higher abundance of orthologous gene groups K18231 and K11959 in the high group compared to both the control and the low group. K18231 showing the largest fold change difference comprises membrane transporters involved in resistance to macrolides. K11959 also comprises transporter genes, linked to the urea. We detected overlap in the functional differences between either intervention group (high or low) and the control. Of the gene orthologues that were significantly different between the high or the low group and the control, four (K00689, K03620, K03605 and K02404) were significantly different in both groups compared to the control. K00689, K03620 and K03605 showed lower abundance in both the low and high group compared to the control, while K02404 was enriched in both groups. K00689 consists of orthologues of dextransucrase, a glycosyltransferase involved in carbohydrate metabolism. Orthologue group K03620 contains a hydrogenase and cytochrome subunit. K03605 is a protease that, by cleaving off a 15 amino-acid peptide, is involved in the terminal processing of a metal-binding hydrogenase. K02404 is a protein necessary for the biosynthesis of the flagellum, and may be involved in translocation of the flagellum.

### Antibiotic resistance genes are significantly more abundant in the high intervention group at day 42

**Fig. 4:**
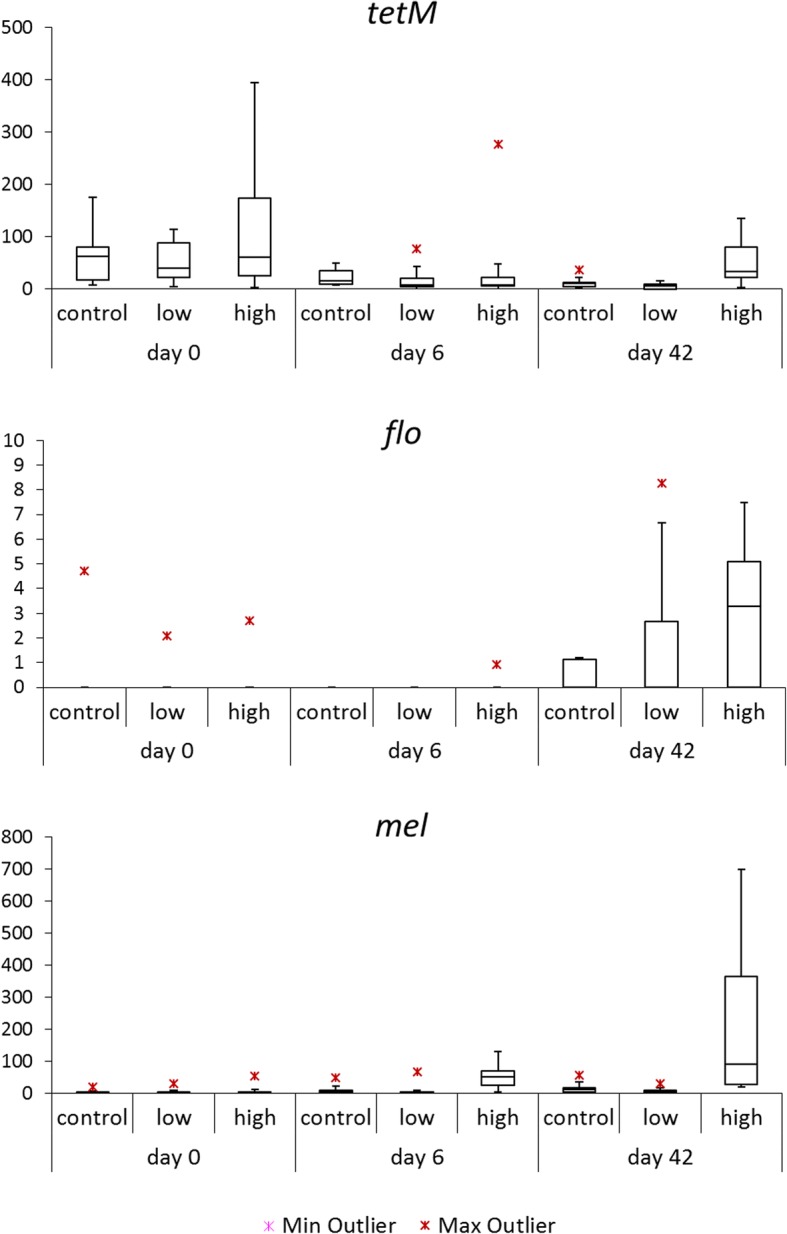
Boxplots showing normalized read abundance of the antibiotic resistance genes *tetM flo and mel* for each group at different time points. Gene abundance is based on metagenomic data. Antibiotic resistance genes were identified for having a significantly higher abundance in the metagenomic database in the treatment group compared to the control group

Antibiotic resistance (AbR) genes in the calf fecal microbiota were identified by aligning the metagenomic sequences of the calf fecal samples taken at day 0, 6 and 42 against the CARD database, a curated database of antibiotic resistance genes. Of the 532 antibiotic resistance genes identified, seven genes showed a significant difference in their relative abundance between either intervention group and the control group in at least one time point. Of these seven genes, two AbR genes had lower and five AbR genes had higher abundance levels in one of the intervention groups compared to the control (Table S3). Within the group of five genes with increased abundance, two genes conferred resistance to florfenicol (*flo/floR*), two genes represented macrolide resistance genes (*mel*), and one conferred resistance to tetracycline (*tetM*). The seventh gene identified by statistical comparison was RNA polymerase beta-prime chain *rpoB*, part of the CARD database as target for rifamycin. The normalized read abundances of the *mel*, *tetM* and *flo* genes in the three groups at the three time points is plotted in Fig. 4.

Compared to the control group, the *tetM* tetracycline normalized gene abundance was found to be significantly higher in the therapeutic OTC-high group (*P* < 0.05). The median normalized gene counts at day 42 were for the control group 10.7, for the OTC-low group 5.3 while for the OTC-high group this was 32.9. It was noted that at day 0, generally the normalized *tetM* gene counts were higher compared to other timepoints analysed. Median normalized *tetM* gene count was 48 at baseline and no significant difference in abundance was detected between groups. At day 6, the median normalized *tetM* gene count was 11,5.

For the *mel* gene encoding a macrolide efflux pump, significantly (P < 0.05) higher gene counts were found for the OTC-high group. This was most pronounced at day 42. While the median gene counts at baseline were low (between 0 and 2.6), at day 6 the median gene count for the OTC-high group was 51. At day 42, the median gene count was 10.3 for the control group, 5.3 for the OTC-low group, and 90 for the OTC-high group. Considerable variation in the normalized gene counts was observed between calves within the OTC-high group at day 42.

*Flo* and *floR* abundance in general was low. Significant higher levels were found at day 42 in the OTC-high and OTC-low group in compared to the control group. The median levels at day 42 were 0 for the control and 2.9 and 1.0 for the OTC-high and OTC-low group respectively.

### qPCR analysis confirms differences in antibiotic resistance genes abundance between groups

**Fig. 5:**
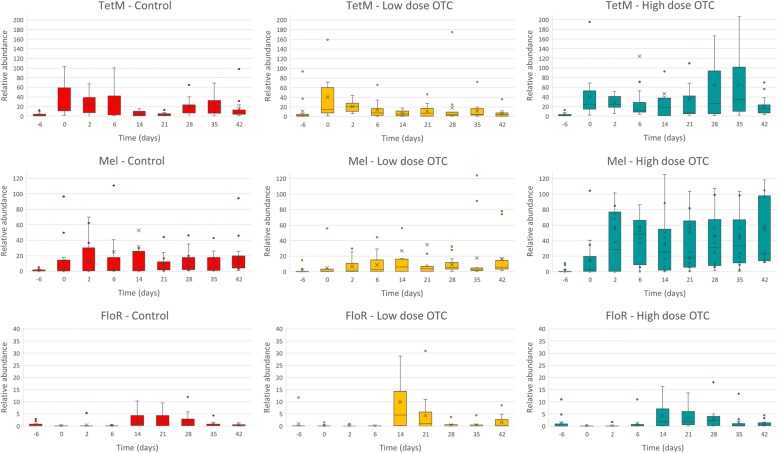
Relative abundance levels of antibiotic resistance genes TetM (upper row), Mel (middle) and floR (lower row) determined by real-time qPCR for each group and time point. The boxes in the plot represent the interquartile ranges, the horizontal lines give the position of the medians, the whiskers indicate the minimum and the maximum values. The dots indicate outliers

To confirm the findings obtained in the resistome metagenomic analysis and to further analyse the dynamic changes in the abundances of the resistance genes identified, quantitative PCR was performed. For this, we retrieved the metagenomic reads mapping to the *mel, tetM and floR* resistance genes and aligned them with corresponding genes from the CARD database. Next, we designed dedicated primers and probes and performed qPCR analysis in all 432 samples collected throughout the intervention. The *mel*, *tetM* and *floR* abundancies were normalized for the total bacterial load as established by broad range 16S ribosomal PCR. The results of the qPCR are showed in Fig. 5. qPCR results confirmed the elevated *tetM* and *mel* abundance in the group receiving therapeutic OTC levels compared to the control in sample acquired at day 42. The relative abundance of *tetM* appeared to be highest between day 28 and day 35 for the OTC-high group. The *tetM* levels did not increase in the samples taken from the OTC-low group. We did detect elevated levels in the samples taken at the start of the intervention, declining to relatively low levels at day 14. The macrolide efflux gene, *mel,* was found to have higher abundance in the therapeutic OTC-high group, compared to the control group. The OTC-low group did not show elevated *mel* levels. *floR* had extremely low levels, near to the qPCR detection limit. It showed a very modest increase at day 14 and remained elevated up until day 28.

## Discussion

In this study we aimed to investigate the effects of long term exposure to a low dose of oxytetracycline (OTC) on the calf fecal microbiota and resistome in vivo in comparison to short term therapeutic OTC treatment and a non-treated control. During the study, calves either received an oral therapeutic dosage of OTC for 5 days, or a low oral dosage of OTC for 7 weeks. Fecal samples were collected at weekly intervals and analysed in a combinatory approach using 16S ribosomal sequencing, shotgun metagenomics, quantitative PCR and UPLC-MS/MS analysis. In the therapeutic group (OTC-high), the fecal OTC levels were at least tenfold higher than the levels established for OTC resistant isolates of 16 μg/ml as reported by the Clinical and Laboratory Standards Institute. In the OTC-low group, OTC levels were hundredfold below these values and were in line with levels detected in contaminated farm environments [12, 13, 33, 34, 36]. Measurable levels of OTC were detected temporarily in the control group despite measures to prevent this. These levels were at least ten-fold lower than those measured in the OTC-low group. The set up thus allowed us to investigate the effects of OTC at therapeutic and sub-therapeutic concentrations and to compare these effects to a group that experienced negligible levels of OTC. However, it should be noted that herd-level application of therapeutic levels of tilmicosin and florfenicol was required during the study for the treatment of respiratory infections.

We observed profound changes in the fecal microbial community structure during the run-in period, between day − 6 and day 0 (baseline), coinciding with a decrease of *Bacteroides* and an increase of *Blautia*, *Bifidobacteria* and *Lactobacillus* species (Fig. 2A). A change in diet may be the main reason for the observed drastic changes in community structure between days − 6 and 0. A more gradual change in fecal microbiota composition was observed between day 0 and day 21, during which *Prevotella* became the dominant genus. After day 21, the fecal microbiota community structure stabilized. Contrary to our expectations, the provision of therapeutic levels of OTC did not result in apparent shifts in calf gut community structure, nor in species diversity, when compared to the control and low-dose conditions. A significant, transient effect of the therapeutic oral dosage of OTC between day 0 and day 2 was determined, as indicated by a larger Bray-Curtis dissimilarity between sample of the same calf taken at these time points (Fig. 3). Whether oral dosage of OTC also impacts other parts of the gastro intestinal tract remains to be determined. Several studies have described the temporal changes in the calf fecal microbiome over time, and suggested ties with changes in the calf diet as well as physiological developments of the gastro intestinal tract [38, 39]. Meale et al. [40] indeed showed that a delayed transition from a high milk diet to an exclusively solid feed diet resulted in a more gradual shift in gut microbiota community structure and functionality. Few studies have investigated the effects of therapeutic application of OTC on the calf fecal microbial community. Oultram et al. [41] presented findings of OTC treatment in seven calves, as part of a prospective cohort study in calves. Although the group size of the study of Oultram et al. [41] was small, discriminant analysis of the five most abundant genera revealed differences in the prevalence of the genus *Lactobacillus* and increased species richness in the treated calves compared to the controls. Antibiotic treatment also related to differences in health status between the animals.

To examine the effects of antibiotic administration on selection for antibiotic resistance genes, we performed metagenomic shotgun sequencing of samples taken at baseline (day 0), at day 6 and at day 42. After mapping metagenomic reads to known antibiotic resistance genes, statistical analysis was used to identify significant differences in gene abundance in any of the intervention groups compared to the control (Fig. 4). The tetracycline resistance gene *tetM*, encoding a ribosome protection protein (RPP) was shown to occur in significantly higher abundance levels in therapeutic group (OTC-high) at day 42. This was not observed for the group receiving the low oral dosage of OTC. Quantitative PCR confirmed elevated levels of *tetM* in the therapeutic group and showed that highest levels were measured at days 28 and 35 (Fig. 5). qPCR revealed no selective enrichment of *tetM* in the group receiving the low OTC dosage. In the group receiving the oral therapeutic dosage of OTC, significantly higher levels of the macrolide resistance gene *mel* were observed. In the group receiving the low dosage of OTC, we did not observe an increase in the levels of *mel*. Quantitative PCR confirmed the observations and showed that elevated levels of *mel* in the oral therapeutic group occurred at all time points after day 6. This coincides with the higher tilmicosin levels throughout the study. Since tilmicosin was given to all calves in all three groups, the significantly higher levels of *mel* resistance in the therapeutic OTC groups may suggest added effects of tetracycline and tilmicosine or multidrug resistance. In various studies on Gram positive clinical isolates, associations have been reported on tetracycline and macrolide resistance [42, 43]. In *Streptococcus pneumoniae*, resistance to macrolides and tetracyclines has linked to a conjugative transposon carrying multiple resistance genes [44, 45]. Phage-mediated transduction of *mefA* and *tetO* has been demonstrated between Streptococcus species [46]. Multidrug resistant *Clostridium perfringens* isolates from water, soil and sewage were shown to be capable of the transfer of *tetM*, *ermB*, *ermQ* and the *mel* orthologue *mefA* genes to the Enterococcus faecalis recipient through independent conjugative processes [47]. Resistome analysis also revealed an significant increase in the abundance of *floR* but its levels remained relatively low.

It has been well established that therapeutic levels of antibiotics can lead to selection of resistant bacteria. The effects of low concentrations of antibiotics, as can be encountered in farming environments, are not well understood. In vitro studies using high sensitive competition assays revealed that selection of resistant bacteria can occur at concentrations of more than hundred-fold below the minimal inhibitory concentration (MIC). For example, competition growth studies with wild type and resistant *Salmonella typhimurium* revealed that the lowest concentration at which novo resistant mutants were selected (minimum selective concentration) was 15 μg/L for tetracycline [6]. Mathematical modelling next showed that competitive advantage would allow such resistant mutants to become dominant in the population of susceptible organisms. Exposure to sub-MIC antibiotic concentrations may therefore be an important mechanisms for the selection and spread of resistance [48]. However, results from studies on the effects of subtherapeutic levels of antibiotics in vivo have not been conclusive, likely reflecting differences in set-up and type of antibiotics used. The effects of low, subtherapeutic concentrations of antibiotics on the fecal microbiota may be more complex as multiple factors drive selection in this ecosystem, including diet, immunity, and physiological conditions. This hampers the direct translation of effects detected in vitro to conditions in vivo. In a study, preweaned calves were fed for 6 weeks with milk spiked with low concentration of an antibiotic mixture of ceftiofur (100 μg/L), penicillin G (5 μg/L), ampicillin (10 μg/L) and OTC (300 μg/L), resulting in higher proportions of antibiotic resistant *Escherichia coli* and a number of genus-level changes in the fecal microbiota composition [49, 50]. Another study, instead, examined the effects of therapeutic (1 g/calf/day for 14 days) and subtherapeutic (10 mg/calf/day for 42 days) levels of neomycin and OTC, and found no changes in the absolute (copies per gram of wet feces), nor the relative (copies per copy of the ribosomal 16S gene) abundance of antibiotic resistance genes corresponding to the tetracycline (*tetC*, *tetG*, *tetW*, and *tetX*), macrolide (*ermB*, *ermF*), and sulfonamide (*sul1*, *sul2*) classes of antibiotics, nor for the class I integron gene, intI1. In that study, the relative abundance of *tetO* was shown to be higher in the therapeutic group [51].

## Conclusions

The aim of this study was to investigate the impact of a long term exposure to a low dose of oxytetracycline on the gut microbiome and resistome of calves in comparison to therapeutic treatment and a non-treated control. Based on 16S rDNA sequence analysis, we observed that antibiotic administration did not cause drastic changes in the composition of the gut microbiome of calves. Within the first 2 days from the start of the intervention, a significant difference was observed between the high dose group and the other groups (control and low dose of antibiotics). However, after day 2, no significant differences were observed between changes occurring in the different groups. Multidimensional scaling analysis showed that time rather than group explained the largest variation in the microbiota between day 0 and day 21, and that after day 21 the microbial community structure appears to have stabilized. Resistome analyses revealed elevated levels of the tetracycline resistance gene *tetM* in the group receiving the oral therapeutic dosage of OTC as well as increased levels of the macrolide efflux gene *mel* in both therapeutic groups. This was not observed in the group receiving the low oral dosage of OTC throughout the 7 weeks study. As in complex microbial communities, multiple factors can act simultaneously to apply selective forces on members of this ecosystem, the net result of subtherapeutic levels of antibiotics are difficult establish. In vitro studies are likely to fall short as they lack some of the key drivers in ecological interactions and probably overestimate the impact of selective agents. In this study, metagenomic sequencing allowed us to establish the dose-dependent impact of antibiotics on the fecal microbial community structure and antibiotic resistance gene abundance in vivo. The unbiased nature of the methodology may be valuable in our efforts to establish minimum selective concentration values for antibiotics.

## Additional files

Additional file 1: **Figure S2.** Spatial distribution of the pens where the calves were housed. The colors indicate the locations of the control group animals (red), the animals receiving a low dose of OTC (yellow), and the animals receiving a high dose of OTC (blue). (PNG 82 kb)

Additional file 2: **Figure S1.** Experiment timeline. The upper part of the figure indicates the time points at which specific antibiotics were administered to the intervention and control groups. The lower part of the figure indicates the time points at which specific analyses were performed. (PNG 36 kb)

Additional file 3: **Table S1.** Primers and probes used in qPCR. **Table S2.** A. Numbers of gene orthologues with significantly (P<0.01) different abundances between groups. Down and up indicate whether the genes are under- or over-represented respectively in one group compared to the other. B. Gene orthologues with significantly (P<0.01) different abundances between groups. Table lists for all significant gene orthologues, the KEGG reference and annotation, and the the Benjamini-Hochberg adjusted p-value (padj). **Table S3.** Resistance genes found in the metagenomic dataset that had a significantly higher abundance (p < 0.05) in one of the intervention groups compared to the control. (DOCX 26 kb)

Additional file 4: **Figure S3.** Bar graph showing the taxonomic composition at the genus level in the control and intervention groups at different time points. The graph shows average abundance values for each group and time point. The “rest” group includes genera detected with a relative abundance lower than 1%. (PNG 53 kb)

Additional file 5: **Figure S4.** Heatmap of the ten operational taxonomic units identified through Canonical Analysis of Principal coordinates (CAP), (A) linked to calf age (time), (B) linked to group differences. (ZIP 20 kb)

Additional file 6: **Figure S5.** Boxplots shoring the Shannon diversity of each group of calves at each time point. The boxes in the plot represent the interquartile ranges, the horizontal lines give the position of the medians, the vertical bars indicate the range. The dots indicate outliers. (PNG 329 kb)

## Abbreviations

CMR: Calf Milk Replacer
CTR: Abbreviation refers to the group of animals receiving not receiving any antibiotics and serving as unexposed control
HGT: Horizontal gene transfer
MDS: Multidimensional scaling
MED: Minimal Entropy Decomposition
MIC: Minimum inhibitory concentration
OTC –high: Abbreviation refers to the group of animals receiving the oral therapeutic dose of oxytetracycline
OTC-low: Abbreviation refers to the group of animals receiving the oral low dose of oxytetracycline
qPCR: Quantitative polymerase chain reaction
UPLC-MS/MS: Ultra performance liquid chromatography - tandem mass spectrometer
