# Supplementary figures and images for: Dose-dependent impact of oxytetracycline on the veal calf microbiome and resistome
Source: BMC Genomics. 2019 Jan 19;20:65. doi: 10.1186/s12864-018-5419-x (PMC6339435; doi:10.1186/s12864-018-5419-x)

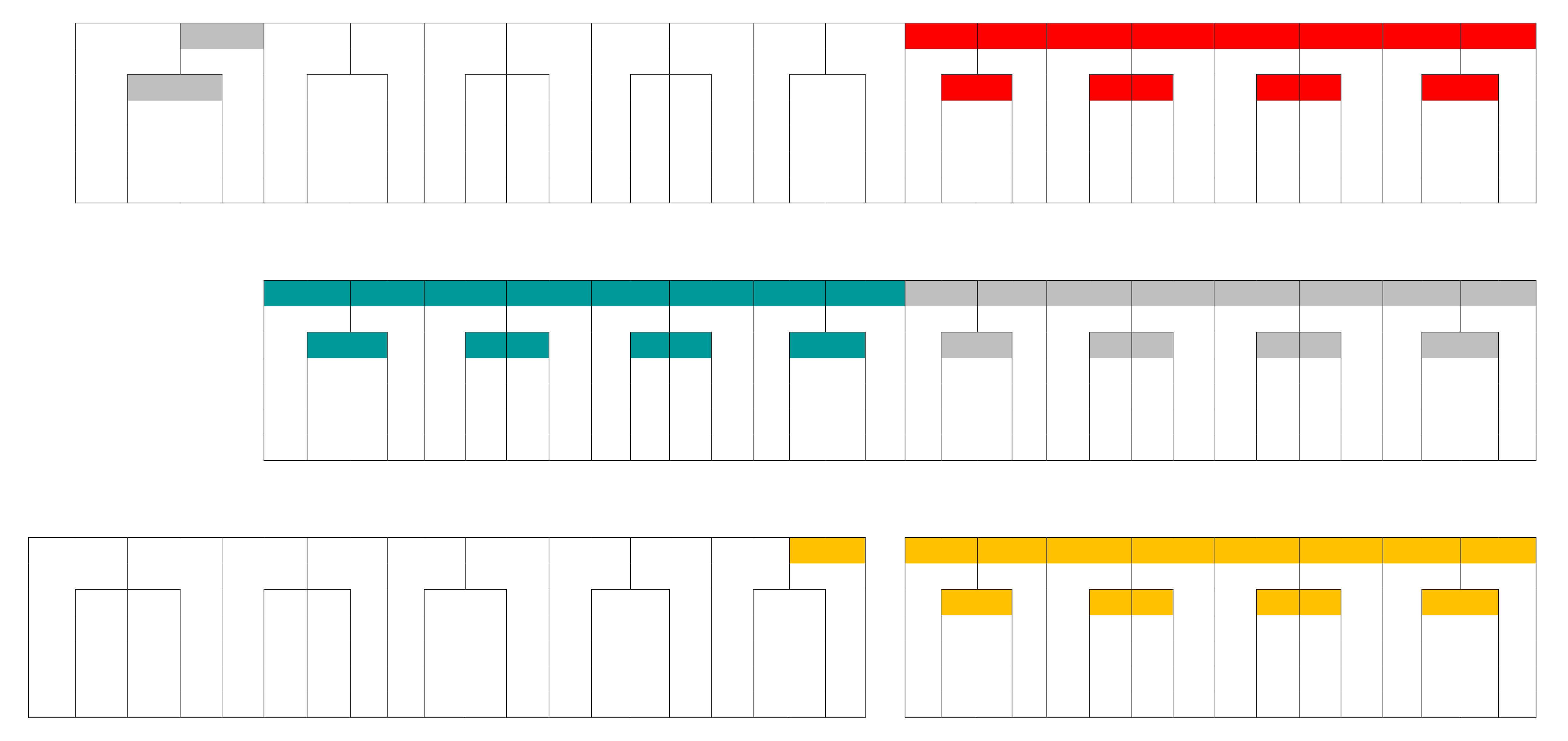

Supplement: Supplementary file 1 — Figure S2. Spatial distribution of the pens where the calves were housed. The colors indicate the locations of the control group animals (red), the animals receiving a low dose of OTC (yellow), and the animals receiving a high dose of OTC (blue). (PNG 82 kb) [file 12864_2018_5419_MOESM1_ESM.png]

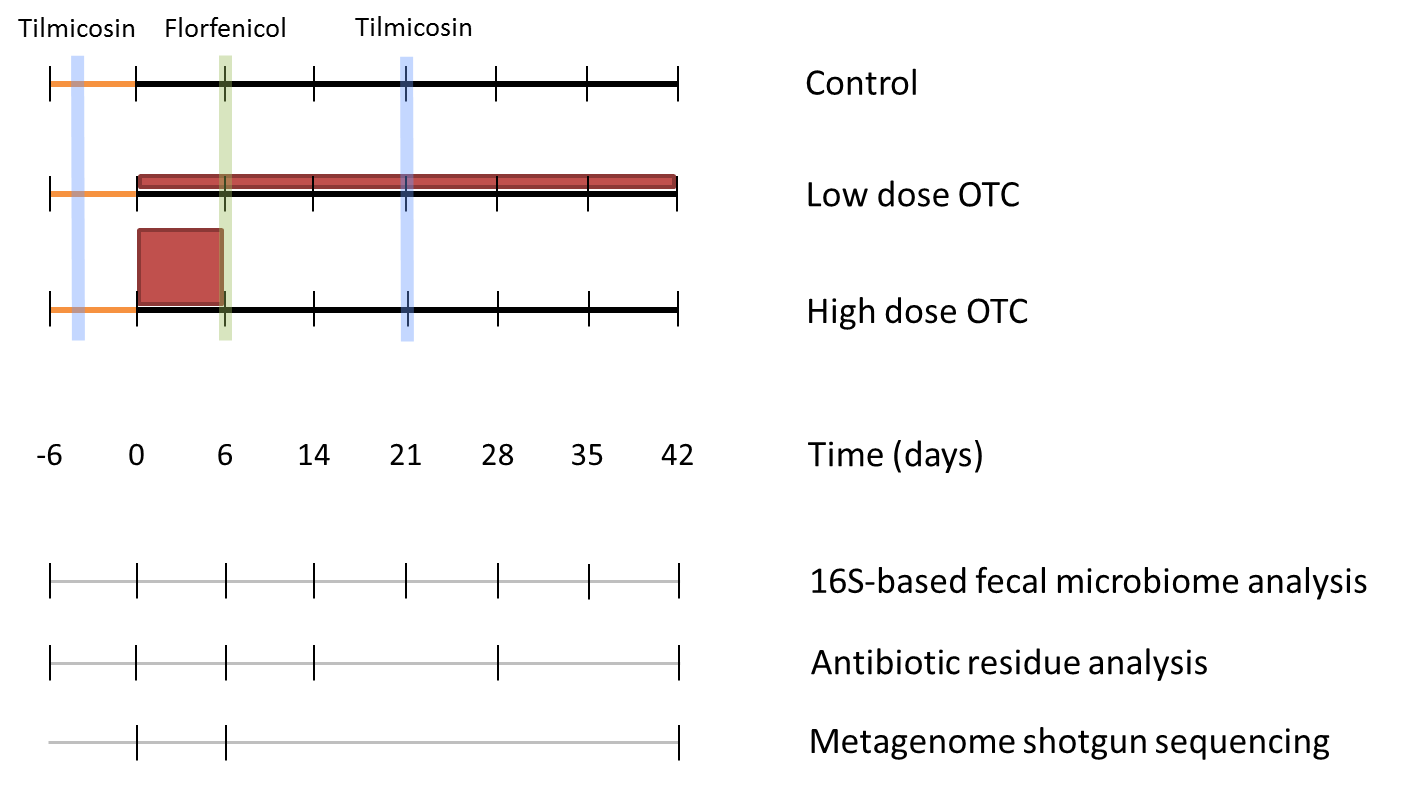

Supplement: Supplementary file 2 — Figure S1. Experiment timeline. The upper part of the figure indicates the time points at which specific antibiotics were administered to the intervention and control groups. The lower part of the figure indicates the time points at which specific analyses were performed. (PNG 36 kb) [file 12864_2018_5419_MOESM2_ESM.png]

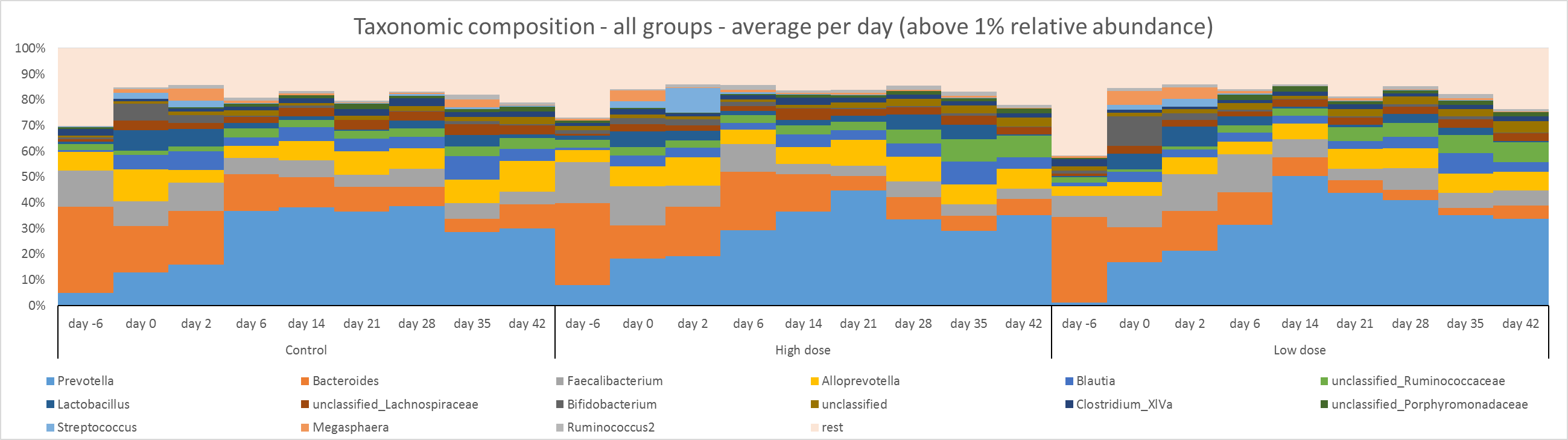

Supplement: Supplementary file 4 — Figure S3. Bar graph showing the taxonomic composition at the genus level in the control and intervention groups at different time points. The graph shows average abundance values for each group and time point. The “rest” group includes genera detected with a relative abundance lower than 1%. (PNG 53 kb) [file 12864_2018_5419_MOESM4_ESM.png]

# Top 10 Time-related sequences

Both negative and positive association

Sequence

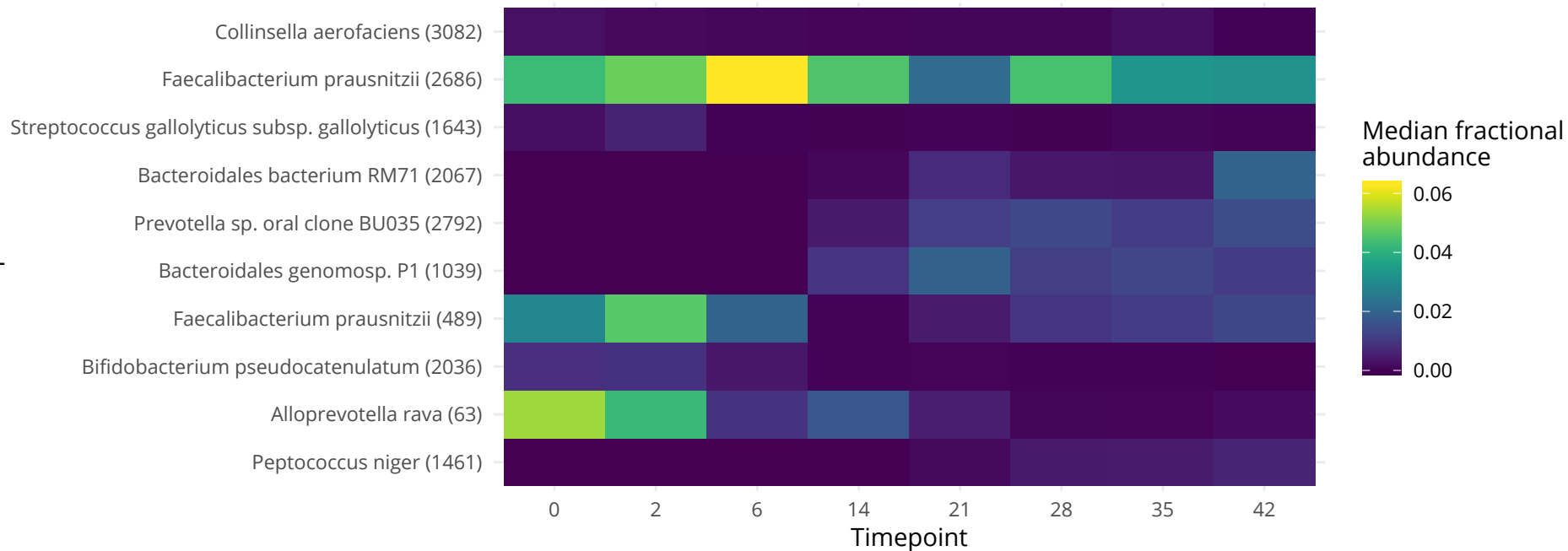

Supplement: Supplementary file 5 — Figure S4. Heatmap of the ten operational taxonomic units identified through Canonical Analysis of Principal coordinates (CAP), (A) linked to calf age (time), (B) linked to group differences. (ZIP 20 kb) [file 12864_2018_5419_MOESM5_ESM.zip › Figure S4A cap.time.heatmap.pdf]

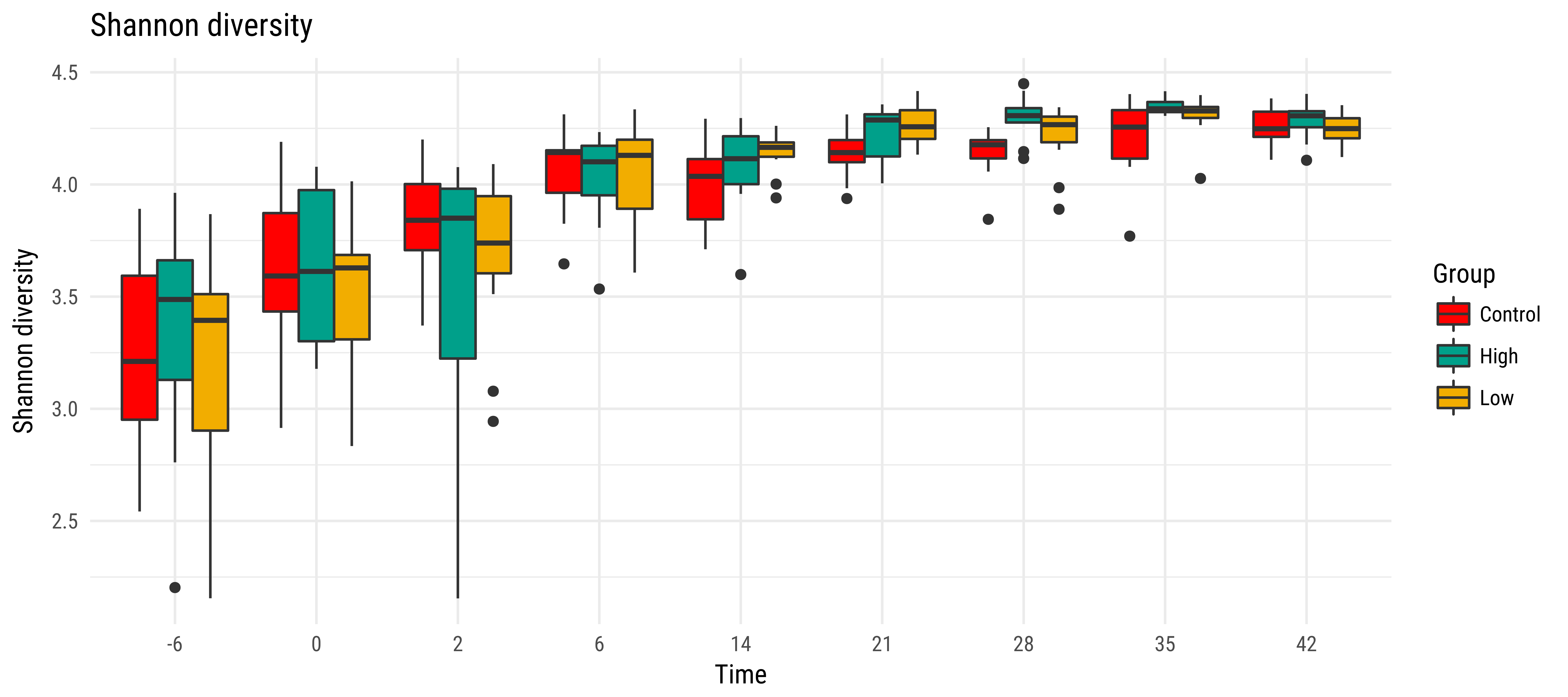

Supplement: Supplementary file 6 — Figure S5. Boxplots shoring the Shannon diversity of each group of calves at each time point. The boxes in the plot represent the interquartile ranges, the horizontal lines give the position of the medians, the vertical bars indicate the range. The dots indicate outliers. (PNG 329 kb) [file 12864_2018_5419_MOESM6_ESM.png]
