# Supplementary material for: Dose-dependent impact of oxytetracycline on the veal calf microbiome and resistome
Source: BMC Genomics. 2019 Jan 19;20:65. doi: 10.1186/s12864-018-5419-x (PMC6339435; doi:10.1186/s12864-018-5419-x)
Supplement: Supplementary file 3 — Table S1. Primers and probes used in qPCR. Table S2. A. Numbers of gene orthologues with significantly (P<0.01) different abundances between groups. Down and up indicate whether the genes are under- or over-represented respectively in one group compared to the other. B. Gene orthologues with significantly (P<0.01) different abundances between groups. Table lists for all significant gene orthologues, the KEGG reference and annotation, and the the Benjamini-Hochberg adjusted p-value (padj). Table S3. Resistance genes found in the metagenomic dataset that had a significantly higher abundance (p < 0.05) in one of the intervention groups compared to the control. (DOCX 26 kb) [file 12864_2018_5419_MOESM3_ESM.docx]

**Table S1**. Primers and probes used in qPCR

| **Target** |  | **Sequence** |
| --- | --- | --- |
| **16S rDNA** | Forward | CGA AAG CGT GGG GAG CAA A |
|  | Reverse | GTT CGT ACT CCC CAG GCG G |
|  | Probe | 6-FAM - ATT AGA TAC CCT GGT AGT CCA - MGB |
| ***tetM*** | Forward | Fw: CCC TGT TAG TAC CCC AGC AGA T |
|  | Reverse | Rv: CCA GCT TTT TTT AAR ACT TGT TCC A |
|  | Probe | Probe: 6-FAM – ATG CTT GCT CCT ATT GTA T - MGB |
| ***mel*** | Forward | CCA AGC TGC AGA ATA CGA ACA A |
|  | Reverse | CCG CAG CCC TTT CCA AT |
|  | Probe | VIC - ATT GCG GAA CGT GCC - MGB |
| ***floR*** | Forward | CCT CGC TTC ACT GGC GAT |
|  | Reverse | GTT CAG GAT GCC GGG CA |
|  | Probe | VIC - ATC TCC CTG TCG TTC CA - MGB |

**Table S2A**. Numbers of gene orthologues with significantly (P<0.01) different abundances between groups. Down and up indicate whether the genes are under- or over-represented respectively in one group compared to the other.

| **day** | **comparison** | **down** | **up** | **total** |
| --- | --- | --- | --- | --- |
|  | low-control | 0 | 0 | 0 |
| **6** | high-control | 0 | 0 | 0 |
|  | high-low | 0 | 1 | 1 |
|  | low-control | 14 | 12 | 26 |
| **42** | high-control | 14 | 12 | 26 |
|  | high-low | 0 | 2 | 2 |

**Table S2B**. Gene orthologues with significantly (P<0.01) different abundances between groups. Table lists for all significant gene orthologues, the KEGG reference and annotation, and the the Benjamini-Hochberg adjusted p-value (padj).

| **Comparison** | **GeneID** | **Annotation data** | **Adj. p value** |
| --- | --- | --- | --- |
| **high-low T=6** | K19172 | DNA sulfur modification protein DndE | 0.0094 |
| **low-control T=42** | K04758 | ferrous iron transport protein A | 0.0018 |
|  | K13694 | lipoprotein Spr | 0.0018 |
|  | K15372 | taurine---2-oxoglutarate transaminase [EC:2.6.1.55] | 0.0035 |
|  | K00689 | dextransucrase [EC:2.4.1.5] | 0.0044 |
|  | K18303 | multidrug efflux pump | 0.0044 |
|  | K01082 | 3'(2'), 5'-bisphosphate nucleotidase [EC:3.1.3.7] | 0.0049 |
|  | K19540 | fructoselysine transporter | 0.0052 |
|  | K03620 | Ni/Fe-hydrogenase 1 B-type cytochrome subunit | 0.0052 |
|  | K03169 | DNA topoisomerase III [EC:5.99.1.2] | 0.0055 |
|  | K00036 | glucose-6-phosphate 1-dehydrogenase [EC:1.1.1.49 1.1.1.363] | 0.0055 |
|  | K03605 | hydrogenase maturation protease [EC:3.4.23.-] | 0.0055 |
|  | K04257 | olfactory receptor | 0.0055 |
|  | K03151 | thiamine biosynthesis protein ThiI | 0.0055 |
|  | K02824 | uracil permease | 0.0055 |
|  | K00939 | adenylate kinase [EC:2.7.4.3] | 0.0057 |
|  | K02404 | flagellar biosynthesis protein FlhF | 0.0057 |
|  | K03710 | GntR family transcriptional regulator | 0.0057 |
|  | K18581 | unsaturated chondroitin disaccharide hydrolase [EC:3.2.1.180] | 0.0057 |
|  | K00803 | alkyldihydroxyacetonephosphate synthase [EC:2.5.1.26] | 0.0074 |
|  | K05879 | dihydroxyacetone kinase, C-terminal domain [EC:2.7.1.-] | 0.0078 |
|  | K06727 | Fc receptor-like 1 [HSA:115350] | 0.0078 |
|  | K01424 | L-asparaginase [EC:3.5.1.1] | 0.0078 |
|  | K11189 | phosphocarrier protein | 0.0078 |
|  | K00652 | 8-amino-7-oxononanoate synthase [EC:2.3.1.47] | 0.0088 |
|  | K01689 | enolase [EC:4.2.1.11] | 0.0088 |
|  | K03407 | two-component system, chemotaxis family, sensor kinase CheA [EC:2.7.13.3] | 0.0088 |
| **high-control T=42** | K19172 | DNA sulfur modification protein DndE | 1.5E-06 |
|  | K18231 | macrolide transport system ATP-binding/permease protein | 1.5E-06 |
|  | K03605 | hydrogenase maturation protease [EC:3.4.23.-] | 0.0004 |
|  | K18197 | rhamnogalacturonan endolyase [EC:4.2.2.23] | 0.0005 |
|  | K01218 | mannan endo-1,4-beta-mannosidase [EC:3.2.1.78] | 0.0006 |
|  | K06610 | MFS transporter, SP family, inositol transporter | 0.0007 |
|  | K02404 | flagellar biosynthesis protein FlhF | 0.0007 |
|  | K00689 | dextransucrase [EC:2.4.1.5] | 0.0009 |
|  | K03146 | thiamine thiazole synthase | 0.0015 |
|  | K03406 | methyl-accepting chemotaxis protein | 0.0018 |
|  | K12343 | 3-oxo-5-alpha-steroid 4-dehydrogenase 1 [EC:1.3.1.22] | 0.0033 |
|  | K16922 | putative peptide zinc metalloprotease protein | 0.0041 |
|  | K03620 | Ni/Fe-hydrogenase 1 B-type cytochrome subunit | 0.0041 |
|  | K13051 | beta-aspartyl-peptidase (threonine type) [EC:3.4.19.5] | 0.0041 |
|  | K11751 | 5'-nucleotidase / UDP-sugar diphosphatase [EC:3.1.3.5 3.6.1.45] | 0.0041 |
|  | K13541 | cobalt-precorrin 5A hydrolase / precorrin-3B C17-methyltransferase [EC:3.7.1.12 2.1.1.131] | 0.0041 |
|  | K11959 | urea transport system substrate-binding protein | 0.0048 |
|  | K03415 | two-component system, chemotaxis family, response regulator CheV | 0.0048 |
|  | K00556 | tRNA (guanosine-2'-O-)-methyltransferase [EC:2.1.1.34] | 0.005 |
|  | K00986 | RNA-directed DNA polymerase [EC:2.7.7.49] | 0.0054 |
|  | K11960 | urea transport system permease protein | 0.0054 |
|  | K15856 | GDP-4-dehydro-6-deoxy-D-mannose reductase [EC:1.1.1.281] | 0.0058 |
|  | K00208 | enoyl-[acyl-carrier protein] reductase I [EC:1.3.1.9 1.3.1.10] | 0.0064 |
|  | K02100 | MFS transporter, SP family, arabinose:H+ symporter | 0.0073 |
|  | K05364 | peptidoglycan glycosyltransferase [EC:2.4.1.129] | 0.0073 |
|  | K16055 | trehalose 6-phosphate synthase/phosphatase [EC:2.4.1.15 3.1.3.12] | 0.0084 |
| **high-low T=42** | K18231 | macrolide transport system ATP-binding/permease protein | 1.2E-06 |
|  | K11959 | urea transport system substrate-binding protein | 0.0031 |

**Table S3**. Resistance genes found in the metagenomic dataset that had a significantly higher abundance (p < 0.05) in one of the intervention groups compared to the control.

|  | **Gene** | **Definition** | **Accession** | **Organism** |
| --- | --- | --- | --- | --- |
| >AY034138.1.gene10 | floR | floR is a plasmid or chromosome-encoded chloramphenicol exporter | ARO:1000001 ARO:3002705 | [Vibrio cholerae MO10] |
| >AF274302.1.gene5 | mel | Mel, a homolog of MsrA, is an ABC transporter associated with macrolide resistance. | ARO:1000001 ARO:3000616 | [Streptococcus pneumoniae] |
| NC_012469.1.7685970 SPT_1925. | mel | SPT_1925 encodes Mel protein. Mel, a homolog of MsrA, is an ABC transporter associated with macrolide resistance. | ARO:1000001 ARO:3000616 | [Streptococcus pneumoniae Taiwan19F-14] |
| AF261825.2.gene36 flo | flo | The Flo transporters can be plasmid- or chromosome-encoded. flo is an important determinant of Florfenicol resistance in animal isolates of *Escherichia coli* but is also found in human pathogens (e.g. *Salmonella enterica* and *Vibrio cholerae*). | ARO: 3003963  ARO:1000001 | [Salmonella enterica subsp. enterica serovar Typhimurium] |
| AM180355.1.gene636 | tetM | TetM is a ribosomal protection protein that confers tetracycline resistance. It is found on transposable DNA elements and its horizontal transfer between bacterial species has been documented. | ARO:1000001 ARO:3000186 | [Clostridium difficile 630] |
