# Supplementary material for: Dose-dependent impact of oxytetracycline on the veal calf microbiome and resistome
Source: BMC Genomics. 2019 Jan 19;20:65. doi: 10.1186/s12864-018-5419-x (PMC6339435; doi:10.1186/s12864-018-5419-x)
Supplement: Supplementary file 5 — Figure S4. Heatmap of the ten operational taxonomic units identified through Canonical Analysis of Principal coordinates (CAP), (A) linked to calf age (time), (B) linked to group differences. (ZIP 20 kb) [file 12864_2018_5419_MOESM5_ESM.zip › Figure S4B cap.group.heatmap.pdf]

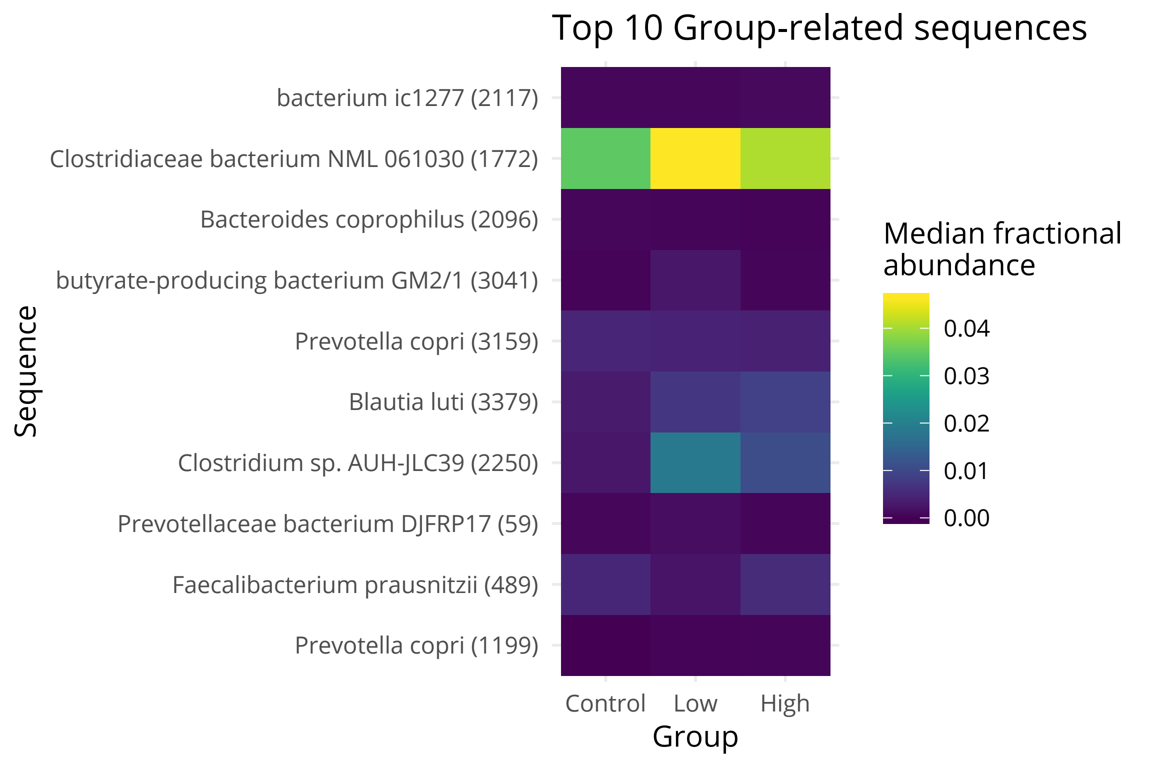
Top 10 Group-related sequences
Sequence
bacterium ic1277 (2117)
Clostridiaceae bacterium NML 061030 (1772)
Bacteroides coprophilus (2096)
butyrate-producing bacterium GM2/1 (3041)
Prevotella copri (3159)
Blautia luti (3379)
Clostridium sp. AUH-JLC39 (2250)
Prevotellaceae bacterium DJFRP17 (59)
Faecalibacterium prausnitzii (489)
Prevotella copri (1199)
Control
Low
High
Group
Median fractional abundance
0.04
0.03
0.02
0.01
0.00
